# Pharmacological investigations of new galantamine peptide esters

**DOI:** 10.1080/13102818.2014.901685

**Published:** 2014-06-04

**Authors:** Dobrina Tsvetkova, Nikolai Danchev, Irina Nikolova, Danka Obreshkova

**Affiliations:** ^a^ Department of Pharmaceutical Chemistry, Faculty of Pharmacy, Medical University of Sofia, Sofia, Bulgaria; ^b^ Department of Pharmacology, Pharmacotherapy and Toxicology, Faculty of Pharmacy, Medical University of Sofia, Sofia, Bulgaria

**Keywords:** galantamine hydrobromide, avoidance learning, Alzheimer's disease

## Abstract

Galantamine hydrobromide (GAL) is a reversible acetylcholinesterase inhibitor, with properties to increase the concentration of acetylcholine in several brain structures. The aim of this study is to determine the effect of new galantamine peptide esters: 3,4-dichlorophenyl-alanil-leucil-glycine-galantamine (GAL-LEU) and 3,4-dichlorophenyl-alanil-valil-glycine-galantamine (GAL-VAL), on locomotor activity in mice and cognitive processes in experimental model of learning and memory in rats. The results showed that per oral administration of GAL-LEU in a dose of 3 mg per kg improved the cognitive processes by increasing the conditional avoidances and learning ability after the 5th day of application and preserved the memory at the 12th day of the study.

## Introduction

Galantamine is an acetylcholinesterase inhibitor [1] with antioxidant and neuroprotective properties for the treatment of different neurodegenerative disorders, including dementia of Alzheimer's type.[2]

The effects of galantamine are linked to: (1) its ability to inhibit acetylcholinesterase, an enzyme mediating the hydrolytic cleavage of acetylcholine and termination of its synaptic actions; (2) its function as a positive allosteric modulator of acetylcholine as agonist of α-7-nicotinic receptors.[3,4]

Galantamine exhibits benefits in cognition behaviour in rats,[5,6] mice [7–9] and rabbits [10,11] with cognitive deficits. Experiments show that administration of galantamine on spontaneous hypertensive rats with insomnia improves short-time memory and increases the efficacy of action and mobility. It might be proposed that the drug can delay the memory decline when it is used by patients with a combination of dementia, hypertension and insomnia.[12] Erkinjuntti et al. [13] describe that in clinical trials with patients with vascular disease and Alzheimer's plus cerebrovascular disease, galantamine enhances cognitive function, behaviour, functional ability and activities. The drug administered in 8 mg/day over 3–6 months reveals statistically significant improvement in mildly cognitively impaired people.[14] Galantamine maintains performance in all domains of Alzheimer's disease: cognition, function, behaviour.[15] Almost 80% of patients, continuously receiving galantamine for up to 36 months demonstrate cognitive benefits.[16]

Galantamine esters newly synthesized by Prof. Vesenkov [17] that contain peptide fragments in the sixth position of the galantamine molecule: 3,4-dichlorophenyl-alanil-leucil-glycine-galantamine and 3,4-dichlorophenyl-alanil-valil-glycine-galantamine; were analysed to determine their influence after per oral administration in different doses on: (1) locomotor activity in mice; (2) cognitive processes in experimental model of learning and memory in rats.

## Materials and methods

### Materials

The galantamine peptide esters: 3,4-dichlorophenyl-alanil-leucil-glycine-galantamine (GAL-LEU) and 3,4-dichlorophenyl-alanil-valil-glycine-galantamine (GAL-VAL) were provided by courtesy of Prof. Vesenkov (Department of Organic Chemistry, University of Chemical Technology and Metallurgy). A molecular grade distilled water and Tween 80 were also used for the study.

### Influence of galantamine peptide esters on locomotor activity in mice

Twenty-five male white mice, line H (body weight 25–35 g) were divided into five groups (*n* = 5): a control group treated with distilled water – 1 ml/100 g; a group treated with GAL-LEU, 2 mg/kg; a group with GAL-LEU, 6 mg/kg; a group with GAL-VAL, 2 mg/kg; a group with GAL-VAL, 6 mg/kg. The determination of the locomotor activity of mice was carried out in Ugo Basile Biological Research (Milan, Italy) apparatus, which gives an account of locomotor activity of animals on the basis of capacitive method. A relationship of dependence time to conditional units of locomotor activity was built according to the obtained results. The solutions were prepared *ех tempore* with distilled water, by using the superficial compound Tween 80.

### Influence of galantamine peptide esters on learning and memory test in rats

Thirty male Wistar rats (body weight 150–200 g) were divided into six groups (*n* = 5): Control 1 and Control 2 groups treated with distilled water; a group treated with GAL-LEU, 1 mg/kg; a group with GAL-LEU, 3 mg/kg; a group with GAL-VAL, 1 mg/kg; a group with GAL-VAL, 3 mg/kg. The solutions were prepared *ех tempore* with distilled water, by using the superficial compound Tween 80.

The dynamic of learning and memory was examined by two-way avoidance learning method in “shuttle box” of Ugo Basile (Italy). The apparatus was divided into two segments, divided by a barrier, giving an opportunity to the animals for avoidance by crossing to the opposite side.

The animals were treated orally for 5 days with the respective solutions in a volume of 0.5 ml/100 g body weight (BW), 30 min before the learning test: 20 stimulation, including conditional (85 dB and light 15W) and unconditional (electric stimulation 60V, 2А, 6Hz). The conditional stimulations for 3.5 s were given primary. If the animal did not pass over the opposite segment in the following 3.5 s (40 conditional units), the conditional signals were supported by electric stimulation floor (40 conditional units). If avoidance did not occur the stimulations were stopped. The statistical analysis of the experimental results was done by *t*-test of Student–Fisher (confidence possibility *p* ≤ 0.05).

## Results and discussion

### Influence of galantamine peptide esters on locomotor activity in mice

**Figure 1.**
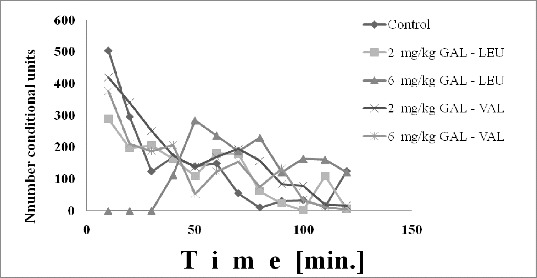
Effect of GA-LEU and GAL-VAL on locomotor activity in mice.

The experimental results from the study of the influence of the examined compounds on locomotor activity in mice are illustrated in Figure 1.

GAL-LEU in a dose of 2 mg/kg did not induce effect on locomotor activity, while the dose of 6 mg/kg induced an increase of locomotion from 50 to 110 min from the start of the experiment. Both tested doses of GAL-VAL did not influence the locomotor activity.

### Influence of galantamine peptide esters on learning and memory test in rats

**Figure 2.**
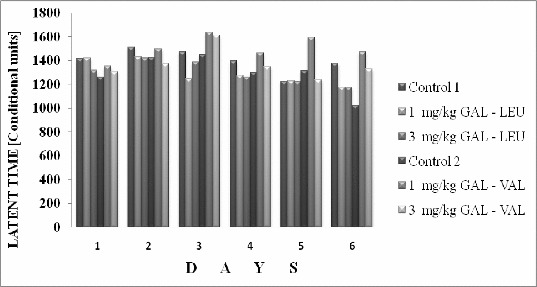
Two-way avoidance learning method: influence of GAL-LEU and GAL-VAL on total time of avoidance in rats.

The results of the influence of the examined peptide esters on the total time of avoidance in rats are illustrated in Figure 2.

**Figure 3.**
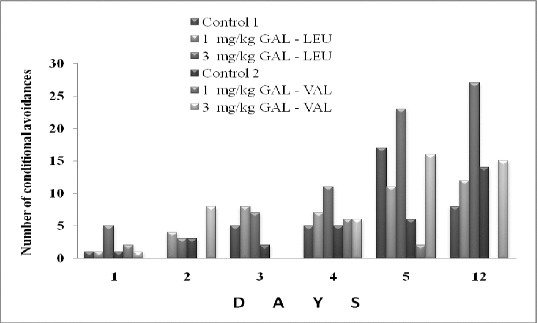
Number of the conditional avoidances of rats after administration of GAL-LEU and GAL-VAL.

The effect of 1 and 3 mg/kg GAL-VAL was similar to the respective control, as a decrease of the total time of avoidance was not observed. In the group of rats treated with 1 and 3 mg/kg GAL-LEU the latent time was decreased and the compound showed a tendency to improve learning. The data for the number of the conditional avoidances of rats as a consequence of the activity of compounds are presented in Figure 3.

The derivative of GAL-LEU in dose 3 mg/kg showed a tendency to improve learning in rats on the 5th day and to preserve memory at the 12th day in the two-way avoidance learning model. The learning and memory of rats were not influenced both by 1 and 3 mg/kg of GAL-VAL during the whole period of the experiment.

In comparison the following effects of galantamine were described by other studies. Following subcutaneous treatment of the drug in doses 3 and 6 mg/kg/day for 15 days enhanced spatial learning in rats.[18] After 16 weeks of alcohol intake and a 2-week pause, rats administered with 2.5 mg/kg/day intraperitonealy (IP) galantamine showed an improved speed of learning and short-term memory in the shuttle box test.[19] Galantamine improved cognitive functions in a model of hypoxia in rats by increasing the number of avoidances during the learning session.[20] A dose of 3 mg/kg galantamine facilitated learning in young rabbits and reversed the deleterious effects of mecamylamine on learning.[21] Subcutaneous treatment of mice for 2 months with 1.3 mg/kg/day galantamine significantly improved spatial accuracy.[16] A treatment of 3 mg/kg galantamine showed ameliorating effect on methamphetamine-induced recognition memory impairment in mice.[22] The combined treatment with galantamine and risperidone both at 0.05 mg/kg showed a synergistic effect to reverse the phencyclidine-induced cognitive impairment in mice.[23]

## Conclusions

Following administration per os on mice, the locomotor activity in comparison with the control group was increased for GAL-LEU in a dose of 6 mg/kg that induced an increase of locomotion from 50 to 110 min from the start of the experiment. Both tested doses of GAL-VAL and 2 mg/mg GAL-LEU did not influence the locomotor activity.

The ester GAL-LEU in a dose of 3 mg/kg affected the dynamics of learning on the 5th day and memory for the learned on the 12th day. However, it may be considered that the effect is related to the central nervous system (CNS), as the increase of the locomotor activity was low and not significant.
